# Cereal fiber improves blood cholesterol profiles and modulates intestinal cholesterol metabolism in C57BL/6 mice fed a high-fat, high-cholesterol diet

**DOI:** 10.29219/fnr.v63.1591

**Published:** 2019-02-25

**Authors:** Shufen Han, Wei Zhang, Ru Zhang, Jun Jiao, Chunling Fu, Xing Tong, Weiguo Zhang, Liqiang Qin

**Affiliations:** 1 Department of Nutrition and Food Hygiene, Jiangsu Key Laboratory of Preventive and Translational Medicine for Geriatric Disease, School of Public Health, Soochow University, Suzhou, China; 2 Suzhou Maternal and Child Health Care and Family Planning Service Center, Suzhou, China; 3 Independent Scientist, Irving, TX, USA

**Keywords:** cereal fiber, cholesterol profiles, cholesterol metabolism, intestine, mice

## Abstract

**Background:**

Dietary intake of cereal fiber has been reported to benefit lipid metabolism through multiple mechanisms. The present study aimed to discover the potential mechanisms by which cereal fiber could modify the intestinal cholesterol metabolism.

**Design:**

Male C57BL/6 mice were fed a reference chow (RC) diet; high-fat, high-cholesterol (HFC) diet; HFC plus oat fiber diet; or HFC plus wheat bran fiber diet for 24 weeks. Serum lipids were measured by enzymatic methods. Western blot was used to determine the protein expressions involved in intestinal cholesterol metabolism.

**Results:**

Our results showed that HFC-induced elevations of serum triglycerides, total cholesterol, and low-density lipoprotein cholesterol were normalized in both groups that received cereal fiber. At the protein level, compared with the HFC diet group, the two cereal fibers, especially the oat fiber, significantly increased the protein expression of peroxisome proliferator-activated receptor alpha, liver X receptor alpha, sterol regulatory element-binding protein (SREBP) 2, low-density lipoprotein receptor, adenosine triphosphate (ATP)-binding cassette A1, and ATP-binding cassette G1, while decreasing the protein expression of Niemann-Pick C1-like protein 1, SREBP-1, fatty acid synthase, and acetyl-coenzyme A carboxylase, which were involved in intestinal cholesterol metabolism.

**Conclusion:**

Taken together, increased intake of cereal fiber improved blood cholesterol profiles and increased the intestinal cholesterol efflux and cholesterol clearance in C57BL/6 mice fed a HFC diet. Oat fiber had a stronger effect than wheat bran fiber on cholesterol metabolism by modulating the PPARα, LXRα, and SREBP signaling pathways.

## Popular scientific summary

- Cereal fiber can decrease intestinal cholesterol absorption, and increase intestinal cholesterol efflux and clearance by modulating the PPARa, LXRa and SREBP signaling pathways, in order to reduce blood cholesterol levels in C57BL/6 mice fed a high-fat, high-cholesterol diet.
- Oat fiber was more effective on improving intestinal cholesterol metabolism than wheat bran fiber.

## 

The prevention of cardiovascular disease (CVD) is a key public health priority. Elevated total cholesterol (TC) and low-density lipoprotein cholesterol (LDL-c) levels are one of the main risk factors for developing CVD (1). Reduction of LDL-c is strongly associated with reduced vascular event rates, particularly when that reduction is achieved with statin agents that block the rate-limiting step of cholesterol synthesis (2). In recent years, dietary fiber has been widely prescribed, alone or associated with lipid-lowering therapies, in order to reduce cholesterol levels. Several population investigations demonstrated that increasing intake of dietary fiber can decrease plasma TC and LDL-c (3) and then reduce the risk of CVD (4, 5). Unfortunately, the exact mechanism by which dietary fiber lowers blood LDL-c levels through regulating intestinal cholesterol metabolism is not completely understood.

The intestinal membrane transporter Niemann-Pick C1-like 1 (NPC1L1) plays a pivotal role in intestinal absorption of dietary cholesterol (6). Animal studies have demonstrated that NPC1L1 knockout mice are resistant to high cholesterol diet-induced hypercholesterolemia because of a substantial reduction in intestinal cholesterol absorption (7). Peroxisome proliferator-activated receptor alpha (PPARα) activation is known to inhibit NPC1L1 and induce adenosine triphosphate (ATP)-binding cassette A1 (ABCA1) via upregulation of the cholesterol-dependent liver X receptor alpha (LXRα) (8), thus decreasing intestinal cholesterol absorption and increasing intestinal cholesterol efflux (9, 10).

Our previous investigation showed that cereal fiber can prevent obesity-related liver lipotoxicity and ameliorate lipid profiles by modulating the sterol regulatory element-binding protein 1 (SREBP-1) signal pathway (11); it can improve leptin resistance and sensitivity in mice fed a high-fat, high-cholesterol diet (12). However, the molecular mechanisms of cereal fiber on the intestinal cholesterol metabolism are not fully understood. Accordingly, the present study aimed to explore how (1) the intestinal cholesterol metabolism including cholesterol efflux and cholesterol clearance and (2) the PPARα, LXRα, and SREBP signal pathways were modulated by cereal fiber in mice.

## Materials and methods

### Experimental animals and diets

Seven-week-old male C57BL/6 mice were purchased from SLAC Laboratory Animal Company and were maintained in standard cages in an air-conditioned room (22±2°C) with a 12:12 h light–dark cycle and 60% relative humidity in compliance with the *Guide for the Care and Use of Laboratory Animals* at Soochow University. All procedures of the investigation were in accordance with the principles outlined in *China Practice for the Care and Use of Laboratory Animal*. All possible efforts were made to minimize the suffering and the number of animals used in the present study. The animals had free access to food and water during the whole experimental period. After 14 days of acclimatization to the animal housing facility, mice were randomly assigned to one of four groups: fed with a reference chow (RC, *n* = 10) diet; high-fat, high-cholesterol (HFC, *n =* 10) diet; HFC plus oat fiber (H-oat, *n =* 12) diet; or HFC plus wheat bran fiber (H-wheat, *n =* 12) diet. The RC diet contained 50 mg/1,000 mg cellulose (BW200) and 3.90 kcal/g with 11.5% of calories from fat, and the HFC diet contained 50 mg/866.75 mg cellulose (BW200) and 4.77 kcal/g with 46% of calories from fat; this diet was purchased from Research Diets, Inc. (New Brunswick, NJ, U.S.A., D12451+1% cholesterol). The H-oat diet containing 4.74 kcal/g and H-wheat diet containing 4.75 kcal/g consisted of an HFC diet supplemented with 0.8% oat fiber and 0.8% wheat bran fiber, respectively. Oat fiber was procured from DSM Nutritional Products Ltd. and contained 44% fiber with 22% β-glucan and 22% insoluble fiber, 20% protein, 20% starch, 5% lipids, and so on. Wheat bran fiber was obtained from Aote Food Science and Technology Company and contained 43% fiber with 9% soluble and 34% insoluble fiber, 18% protein, 24% starch, 4.8% lipids, and so on. The two fibers were directly mixed with the HFC diet according to the recipe mentioned, and the feed was formed into strips and dried before feeding. The percentage and type of fat were almost consistent with the HFC diet. The experiment lasted for 24 weeks.

### Sample collection and biochemical analysis

After 24 weeks of the experiment, the mice were fasted overnight and then sacrificed after collecting the blood sample. The serum was separated by centrifugation, subpackaged, and stored at –80°C in a freezer until being assayed. The small intestine tissues were immediately collected, washed three times with 0.9% sodium chloride, dissected into three segments (the duodenum, jejunum, and ileum) according to anatomical structure, frozen in liquid nitrogen, and then stored at –80°C in a freezer for further analysis. Serum levels of TC and triglycerides (TG) were determined by enzyme assay kits from Applygen Technologies, Inc. Serum high-density lipoprotein cholesterol (HDL-c) and LDL-c concentrations were measured using the enzyme standard colorimetric method from Nanjing Jiancheng Bioengineering Institute, following its instructions.

### Western blot analysis

The small intestine tissue samples were lysed in immunoprecipitation lysis buffer (Beyotime, Nantong, China). The lysates were homogenized and centrifuged. The supernatants were collected and the protein concentrations were determined by using a BCA Protein Assay Kit (Beyotime). Equal amounts (30 μg) of ABCA1, ATP-binding cassette G1 (ABCG1), ATP-binding cassette G8 (ABCG8), acetyl-coenzyme A carboxylase (ACC), fatty acid synthase (FAS), low-density lipoproteins receptor (LDLR), LXRα, NPC1L1, PPARα, Sar1B GTPase (Sar1B), scavenger receptor B (SR-B1), SREBP-1, and SREBP-2 were determined by Western blot analysis. Further, the antibodies of ABCA1, ABCG1, ABCG8, ACC, FAS, LDLR, LXRα, NPC1L1, PPARα, Sar1B, SR-B1, SREBP-1, and SREBP-2 were purchased from Abcam (Cambridge, MA, U.S.A.), Cell Signaling Technology (Danvers, MA, U.S.A.), EMD Millipore (Billerica, MA, U.S.A.), or Thermo Fisher Scientific, Inc (Waltham, MA, U.S.A.). Antibody reactivity was detected by Chemiluminescence ECL Detection Systems (EMD Millipore, Billerica, MA, U.S.A.). Subsequently, the intensity of the bands was quantified by densitometry via Gene Tool according to the manufacturer’s instructions (SynGene, Chemi Genius2, PerkinElmer, Wesville, U.S.A.). Beta-actin was used as internal control.

### Statistical analysis

All of the statistical analyses were conducted using SPSS version 17.0 statistical analysis package (SPSS Inc., Chicago, IL, USA). Data are presented as means ± SDs. The significance of difference among the four dietary groups was assessed by analysis of one-way variance (ANOVA), followed by Tukey’s *post hoc* test. Statistical significance was established at *P*-values < 0.05.

## Results

**Fig. 1:**
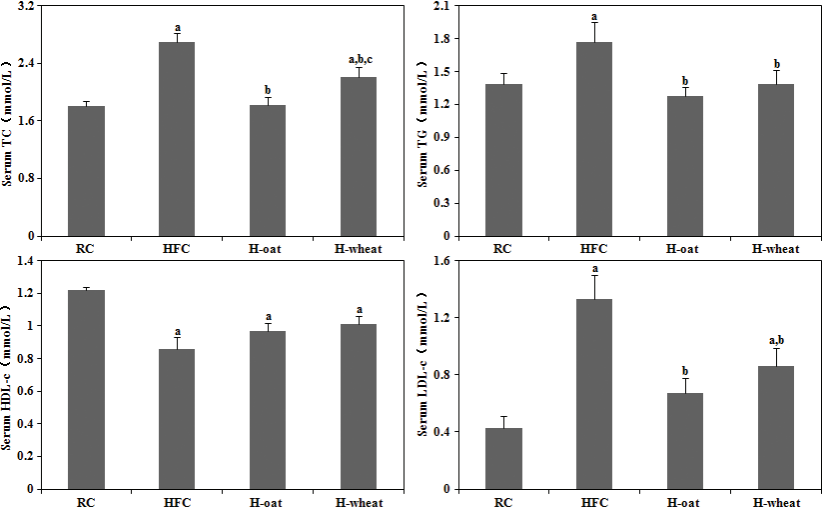
Cereal fiber decreased serum TC, TG, and LDL-c levels without changing HDL-c levels in mice fed a HFC diet. Values are means ± SD (*n* = 10–12). ^a^*P* < 0.05, versus mice fed with RC diet. ^b^*P* < 0.05, versus mice fed with HFC diet. ^c^*P* < 0.05, versus mice fed with H-oat diet. HFC, high-fat, high-cholesterol diet; H-oat, HFC diet plus 0.8% oat fiber; H-wheat, HFC diet plus 0.8% wheat bran fiber; HDL-c,high-density lipoprotein cholesterol; LDL-c, low-density lipoprotein cholesterol; RC, reference diet; TC, total cholesterol; TG, triglyceride.

As shown in Fig. 1, compared with the RC group, the HFC group exhibited an elevation in serum TC (by 48.6%), TG (by 27.3%), and LDL-c (by 209.3%) and a reduction in serum HDL-c (by 29.5%) (*P* < 0.05). The results suggested that the animal model had been built successfully. After 24 weeks being fed cereal fiber, serum TC, TG, and LDL-c were significantly lower in the H-oat and H-wheat groups than in the HFC group (*P* < 0.05) (Fig. 1). The serum HDL-c level showed an increasing trend in the two cereal fiber groups (H-oat, 0.96±0.16 mmol/L; H-wheat, 1.00±0.16 mmol/L), compared with the HFC group (0.86±0.19 mmol/L), but there were no statistically significant differences among the three groups (*P* > 0.05). Furthermore, a significantly lower serum TC level was observed in the H-oat group (1.82±0.39 mmol/L) than that in the H-wheat group (2.21±0.50 mmol/L) (*P* < 0.05). There was no statistically significant difference in other lipid profiles including LDL-c, HDL-c, and TG between the H-oat group and the H-wheat group (*P* > 0.05).

**Fig. 2:**
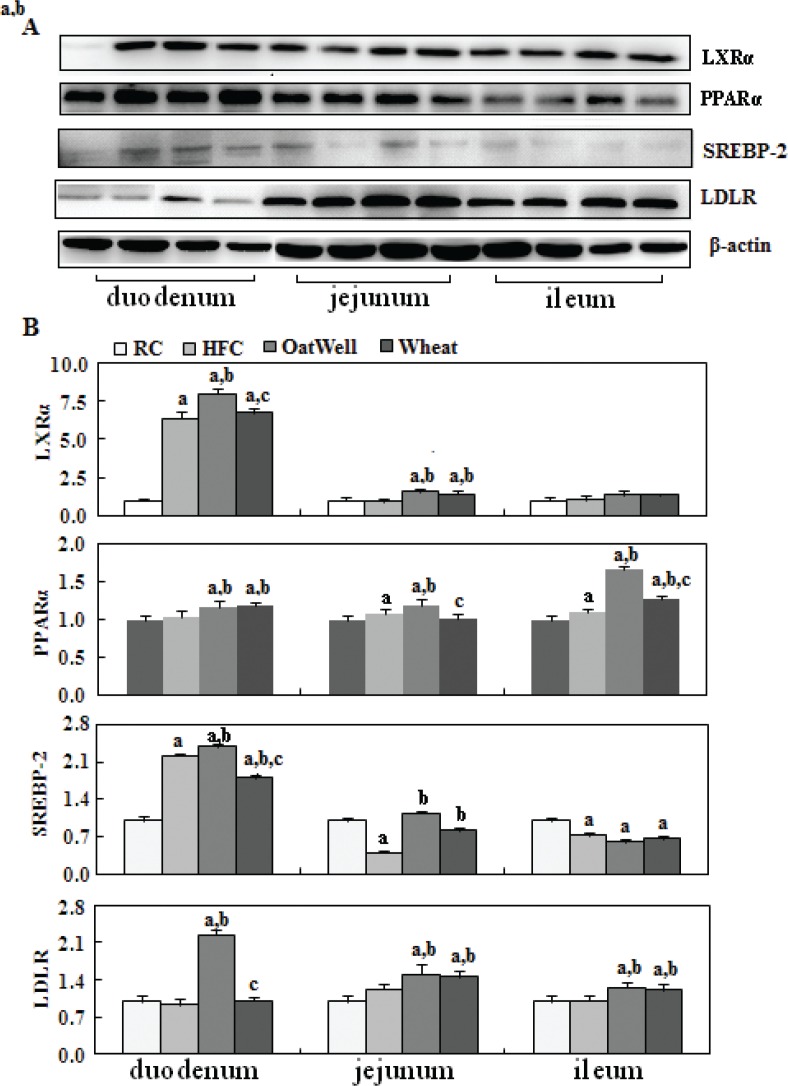
Cereal fiber improved cholesterol metabolism by increasing PPARα, LXRα, SREBP-2, and LDLR expression in the small intestine tissues of mice fed a HFC diet. Values are means and SD (*n* = 6). ^a^*P* < 0.05, versus mice fed with RC diet. ^b^*P* < 0.05, versus mice fed with HFC diet. ^c^*P* < 0.05, versus mice fed with H-oat diet. HFC, high-fat, high-cholesterol diet; H-oat, HFC diet plus 0.8% oat fiber; H-wheat, HFC diet plus 0.8% wheat bran fiber; LDLR, low-density lipoprotein receptor; LXRα, liver X receptor alpha; PPARα, peroxisome proliferator-activated receptor alpha; RC, reference diet group; SREBP-2, sterol regulatory element-binding protein 2.

**Fig. 3:**
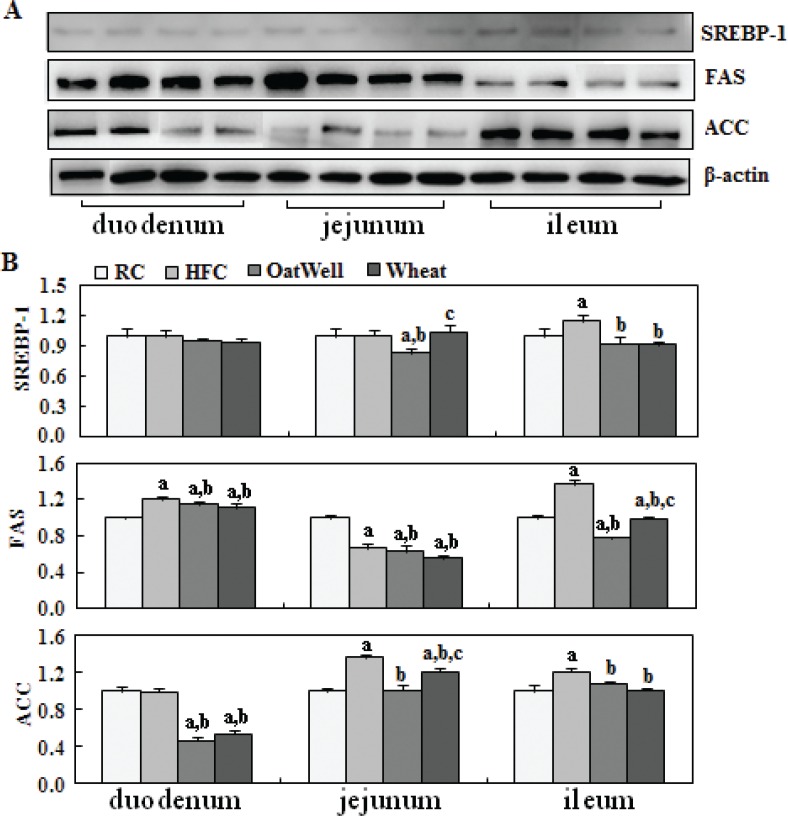
Cereal fiber reduced lipogenesis by inhibiting SREBP-1, FAS, and ACC expression in the small intestine tissues of mice fed a HFC diet. Values are means and SD (*n* = 6). ^a^*P* < 0.05, versus mice fed with RC diet. ^b^*P* < 0.05, versus mice fed with HFC diet. ^c^*P* < 0.05, versus mice fed with H-oat diet. ACC, acetyl-coA carboxylase; FAS, fatty acid synthase; HFC, high-fat, high-cholesterol diet; H-oat, HFC diet plus 0.8% oat fiber; H-wheat, HFC diet plus 0.8% wheat bran fiber; RC, reference diet; SREBP-1, sterol regulatory element-binding protein 1.

To provide molecular evidence for the role of cereal fiber in intestinal cholesterol metabolism, we evaluated the protein expression of PPARα, LXRα, SREBP-2, and LDLR in small intestine tissue samples by Western blot (Fig. 2a). The HFC diet increased LXRα and SREBP-2 expression in the duodenum, compared with the RC group (*P* < 0.05). After oat fiber was administered to the HFC diet-fed C57BL/6 mice for 24 weeks, protein expression of PPARα, LXRα, SREBP-2, and LDLR was considerably upregulated (*P* < 0.05) (Fig. 2b), especially in the duodenum and jejunum. Compared with the HFC group, the H-wheat group had increased protein expression of LXRα, SREBP-2, and LDLR in the jejunum (*P* < 0.05), while only minor changes were observed in the duodenum and ileum. Figure 3 shows the effects of cereal fiber on lipogenesis in small intestine tissue. The HFC diet mainly increased the protein expression of SREBP-1, FAS, and ACC in the ileum, and overexpression was inhibited by cereal fiber supplementation. Compared with the HFC group, both the H-oat and H-wheat groups had decreased protein expressions of FAS and ACC in the duodenum, jejunum, and ileum (*P* < 0.05). In addition, the H-oat group showed decreased protein expression of SREBP-1 in the jejunum and ileum (*P* < 0.05), and the H-wheat group showed decreased protein expression of SREBP-1 in the ileum (*P* < 0.05). By comparison, in the H-oat group, SREBP-1 and ACC in the jejunum, and FAS in the ileum, were much lower that in the H-wheat group. No differences were detected in other factors of the two groups of H-oat and H-wheat (*P* > 0.05).

**Fig. 4:**
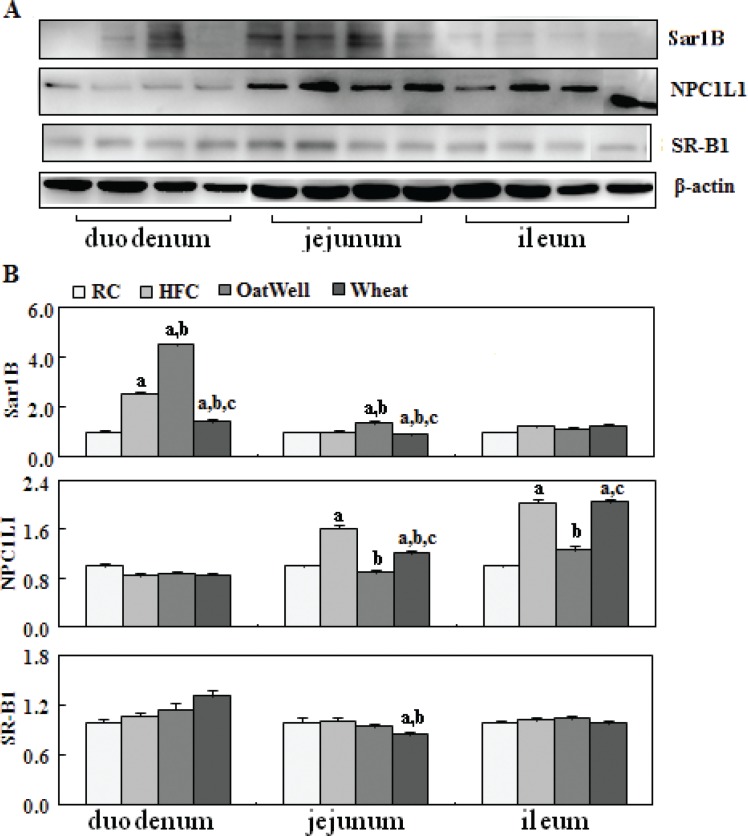
Cereal fiber reduced cholesterol absorption by decreasing NPC1L1 expression and increasing Sar1B expression in the small intestine tissue of mice fed a HFC diet for 24 weeks. Values are means and SDs (*n* = 6). ^a^*P* < 0.05, versus mice fed with RC diet. ^b^*P* < 0.05, versus mice fed with HFC diet. ^c^*P* < 0.05, versus mice fed with H-oat diet. HFC, high-fat, high-cholesterol diet; H-oat, HFC diet plus 0.8% oat fiber; H-wheat, HFC diet plus 0.8% wheat bran fiber; NPC1L1, Niemann-Pick C1-like 1; RC, reference diet; SR-B1, scavenger receptor B.

NPC1L1 played an important role in intestine cholesterol absorption. As shown in Fig. 4, compared with the RC group, the HFC diet increased NPC1L1 expression in the jejunum and ileum (*P* < 0.05), and overexpression was retarded by the two cereal fibers, especially by oat fiber (*P* < 0.05). In addition, compared with the HFC group, the H-oat group showed increased protein expression of Sar1B in the duodenum and jejunum (*P* < 0.05), while the H-wheat group showed a reduced Sar1B protein level in the duodenum (*P* < 0.05). Oat fiber had almost no effect on SR-B1 expression in the small intestine tissue, while wheat bran fiber decreased the expression of SR-B1 in the jejunum.

**Fig. 5:**
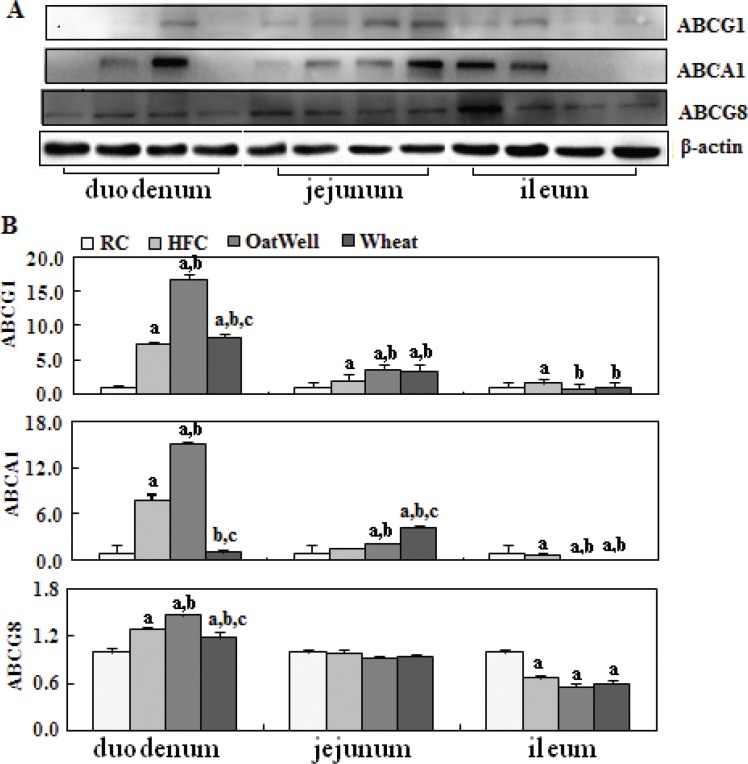
Cereal fiber increased cholesterol efflux by promoting ABCG1 and ABCA1 expression in the small intestine tissue of mice fed a HFC diet. Values are means and SD (*n* = 6). ^a^*P* < 0.05, versus mice fed with RC diet. ^b^*P* < 0.05, versus mice fed with HFC diet. ^c^*P* < 0.05, versus mice fed with H-oat diet. ABCA1, adenosine triphosphate (ATP)-binding cassette A1; ABCG1, ATP-binding cassette G1; ABCG8, ATP-binding cassette G8; HFC, high-fat, high-cholesterol diet; H-oat, HFC diet plus 0.8% oat fiber; H-wheat, HFC diet plus 0.8% wheat bran fiber; RC, reference diet.

Figure 5 showed the role of cereal fiber on cholesterol efflux in the small intestine tissue. Compared with the HFC and RC groups, the H-oat group had increased protein expressions of ABCG1, ABCA1, and ABCG8 (*P* < 0.05), and this phenomenon mainly occurred in the duodenum, while the H-wheat group had slightly increased ABCG1 and ABCA1 protein levels in the jejunum (*P* < 0.05). In the ileum, the two cereal fibers had no effect on the transformed factors involved in cholesterol efflux.

## Discussion

In this study, we explored the role of cereal fiber on blood cholesterol profiles and intestinal cholesterol metabolism in mice fed a high-fat, high-cholesterol diet. The new findings of our present study are as follows: first, cereal fiber can reduce serum LDL-c levels through upregulating SREBP-2 and then increasing the expression of LDLR in the small intestine tissue of C57BL/6 mice (oat fiber mainly in the duodenum and jejunum; wheat bran fiber only in the jejunum). Second, cereal fiber can activate transcription factor PPARα and then upregulate cholesterol sensor LXRα in order to decrease intestinal cholesterol absorption by inhibiting NPC1L1 (oat fiber mainly in the jejunum and ileum; wheat bran fiber only in the jejunum) and increase intestinal cholesterol efflux by inducing ABCA1 and ABCG1 (oat fiber mainly in the duodenum and jejunum; wheat bran fiber only in the jejunum). Third, when these effects were compared, oat fiber was more effective at changing cholesterol metabolism than wheat bran fiber. The respective reductions in TC and LDL-c were 32.3% and 49.6% with oat fiber and 17.8% and 35.3% with wheat bran fiber after 24 weeks of feeding, which is also comparable to the results from other animal studies (13). More beneficial effects of oat fiber on the cholesterol profiles are ascribed to its soluble fiber component, β-glucan. There are many population studies indicating the efficacy of oat with β-glucan in reducing plasma TC and LDL-c (14, 15). Several mechanisms have been proposed for the cholesterol-lowering effect of β-glucan, including 1) increasing the viscosity of intestinal contents and forming an unstirred layer over the intestinal mucosa, which in turn results in reduced absorption of dietary cholesterol (16); 2) reducing hepatic cholesterol synthesis secondary to improved insulin sensitivity (17); 3) inhibiting hepatic cholesterol synthesis through short-chain fatty acids produced by colonic bacteria fermentation of soluble fibers (18); and 4) reducing reabsorption of bile acids to decrease the plasma levels of cholesterol (19). The β-glucan content in oat fiber was 22%, and our previous investigation demonstrated that oat fiber was more effective in improving insulin resistance and increasing insulin sensitivity (11). Intestinal cholesterol absorption efficiency is positively correlated with plasma cholesterol levels (20), and plasma cholesterol level is positively correlated with the incidence of CVD (21). It follows that a reduction of cholesterol absorption in the small intestine tissue should be beneficial in the prevention of CVD. The present study explored the relevant mechanisms.

Transcription factors of the SREBP family have been established as lipid synthetic transcription factors especially for cholesterol and fatty acid synthesis (22), which played an important role in the regulation of cholesterol uptake, efflux, or metabolism (23, 24). *In vivo* animal studies suggest that SREBP-2 regulates the transcription of LDLR for cellular uptake of LDL cholesterol and clearance of plasma cholesterol, whereas SREBP-1 seems to be involved in energy metabolism including fatty acid metabolism (25). Our study demonstrated that mice fed the H-oat diet had significantly higher levels of protein expression for SREBP-2 and LDLR in the duodenum and jejunum segments, compared with those fed the HFC diet. This suggested that oat fiber activated the transcription factor SREBP-2 and upregulated the protein expression of LDLR, which increased intestinal cholesterol removal and decreased plasma cholesterol levels (26). In addition, SREBP-1 and key factors involved in lipogenesis including FAS and ACC were inhibited after two cereal fibers were added to the HFC diet, which reduced the fatty acid synthesis and improved dyslipidemia (27). Moreover, the inhibition mainly appeared in the jejunum and ileum. Our previous study demonstrated that cereal fiber supplementation abrogated obesity-related liver lipotoxicity by modulating SREBP-1 signaling pathways in the liver tissues (11).

Liver X receptors are other important nuclear receptors that function as cholesterol sensors and regulate cholesterol homeostasis (28). A primary function of LXRα as a cholesterol sensor is to maintain cellular cholesterol homeostasis by participating in the process of reverse cholesterol transport (29). LXRα can regulate the expression of genes involved in reverse cholesterol transport, such as ABCA1 and ABCG1, which mediate cholesterol efflux to lipid-poor apolipoproteins (30). In our study, cereal fiber enhanced the protein expression of LXRα (oat fiber mainly in the duodenum and jejunum; wheat bran fiber only in the jejunum), and oat fiber had a stronger effect than wheat bran fiber. We speculated that activated LXRα further promoted intestinal cholesterol efflux through upregulating the expression of transmembrane proteins ABCA1 and ABCG1 (31). In the meanwhile, activated LXRα also increased ABCG8 expression, in order to regulate cholesterol metabolism, efflux, and elimination (32). Our results showed that oat fiber mainly increased the protein expression of ABCA1, ABCG1, and ABCG8 in the duodenum segment. These results suggested that oat fiber can strengthen intestinal cholesterol efflux through activating the LXRα cholesterol sensors. However, wheat bran fiber had little effect on intestinal cholesterol efflux.

Among various proteins involved in the process of intestinal cholesterol absorption, NPC1L1, which is predominantly expressed in the small intestine, plays an important role in intestinal cholesterol absorption (6, 33). Animal studies showed that NPC1L1 was highly expressed in the jejunum and proximal ileum of mice (34). A study demonstrated that activated PPARα is known to inhibit NPC1L1 via upregulation of the cholesterol sensor LXRα (8). Our results showed that oat fiber activated the transcription factor PPARα in the small intestine tissue and then inhibited the protein expression of NPC1L1 in the jejunum and ileum segments, through enhancing the cholesterol sensor LXRα. The effect of wheat bran fiber on inhibiting NPC1L1 expression was minor. This result hinted that oat fiber has a more obvious effect on decreasing intestinal cholesterol absorption than wheat bran fiber. A previous study demonstrated that Sar1B overexpression led to a decrease in NPC1L1 and SR-BI expression (35). In our study, oat fiber upregulated the protein expression of Sar1B in the duodenum and jejunum but had no effect on SR-BI expression. Another investigation also demonstrated that intestinal SR-BI does not impact cholesterol absorption or transintestinal cholesterol efflux in mice (36). However, wheat bran fiber decreased the protein expression of SR-BI in the jejunum. We speculated that wheat bran fiber activated the nuclear receptor LXRα and then downregulated the expression of SR-B1. Cereal fiber, which can reduce intestinal cholesterol absorption through regulating SR-B1, need further research. Unfortunately, the present study did not measure intestinal cholesterol absorption by isotope labeling.

In conclusion, the present study provides further evidences that cereal fiber can improve blood cholesterol profiles and intestinal cholesterol metabolism in C57BL/6 mice fed a high-fat, high-cholesterol diet. The cholesterol-lowering mechanisms of cereal fiber include increasing SREBP-2 to clear away intestinal cholesterol, reducing SREBP-1 to decrease lipid accumulation, and activating transcription factors PPARα and LXRα to increase intestinal cholesterol efflux by upregulating ABCA1 and ABCG1 and decrease intestinal cholesterol absorption by downregulating NPC1L1. Moreover, oat fiber was more effective than wheat bran fiber at ameliorating intestinal cholesterol metabolism by modulating the PPARα, LXRα, and SREBP signaling pathways. Although the modulation of the PPARα, LXRα, and SREBP signaling pathways by cereal fiber is highly assumed to play a pivotal role in ameliorating intestinal cholesterol metabolism, the causal relationship warrants further investigation.

## Conflict of interest and funding

The authors declare no competing financial interest. The present study was financially supported by the National Natural Science Foundation of China (No. 81302425), the 2016 Chinese Nutrition Society Nutrition Research Foundation – DSM Research Fund (No. 2016097B-3), and the Scientific and Technological Projects of Suzhou City (No. SYS2018022).
